# Gut Microbial Composition and Function Are Altered in Patients with Early Rheumatoid Arthritis

**DOI:** 10.3390/jcm8050693

**Published:** 2019-05-16

**Authors:** Yunju Jeong, Ji-Won Kim, Hyun Ju You, Sang-Jun Park, Jennifer Lee, Ji Hyeon Ju, Myeong Soo Park, Hui Jin, Mi-La Cho, Bin Kwon, Sung-Hwan Park, Geun Eog Ji

**Affiliations:** 1 Research Center, BIFIDO Co., Ltd., 23-16, Nonggongdanji-gil, Hongcheon 25117, Korea; tanklov0@snu.ac.kr (Y.J.); bifidopark@bifido.com (M.S.P.); kb@bifido.com (B.K.); 2 Department of Food and Nutrition, Research Institute of Human Ecology, Seoul National University, 1, Gwanak-ro, Gwanak-gu, Seoul 08826, Korea; dhlover1@snu.ac.kr (H.J.Y.); sjpark-90@snu.ac.kr (S.-J.P.); jh1030@snu.ac.kr (H.J.); 3 Division of Rheumatology, Department of Internal Medicine, Seoul St. Mary’s Hospital, College of Medicine, The Catholic University of Korea, 222 Banpo-Daero, Seocho-gu, Seoul 137-701, Korea; shyqw@naver.com (J.-W.K.); poohish@catholic.ac.kr (J.L.); juji@catholic.ac.kr (J.H.J.); 4 Institute of Health and Environment, Graduate School of Public Health, Seoul National University, 1, Gwanak-ro, Gwanak-gu, Seoul 08826, Korea; 5 Rheumatism Research Center, Catholic Research Institute of Medical Science, College of Medicine, The Catholic University of Korea, Seoul 137-040, Korea; iammila@catholic.ac.kr

**Keywords:** rheumatoid arthritis, autoimmune disease, gut microbiome, *Collinsella*, microbial diversity, dysbiosis

## Abstract

Rheumatoid arthritis (RA) is an autoimmune disease characterized by synovial inflammation of the joints and extra-articular manifestations. Recent studies have shown that microorganisms affect RA pathogenesis. However, few studies have examined the microbial distribution of early RA patients, particularly female patients. In the present study, we investigated the gut microbiome profile and microbial functions in early RA female patients, including preclinical and clinically apparent RA cases. Changes in microbiological diversity, composition, and function in each group were analyzed using quantitative insights into microbial ecology (QIIME) and phylogenetic investigation of communities by reconstruction of unobserved states (PICRUSt). The results revealed the dysbiosis due to decreased diversity in the early RA patients compared with healthy subjects. There were significant differences in the microbial distribution of various taxa from phylum to genus levels between healthy subjects and early RA patients. Phylum Bacteroidetes was enriched in early RA patients, while Actinobacteria, including the genus *Collinsella,* was enriched in healthy subjects. Functional analysis based on clusters of orthologous groups revealed that the genes related to the biosynthesis of menaquinone, known to be derived from gram-positive bacteria, were enriched in healthy subjects, while iron transport-related genes were enriched in early RA patients. Genes related to the biosynthesis of lipopolysaccharide, the gram-negative bacterial endotoxin, were enriched in clinically apparent RA patients. The obvious differences in microbial diversity, taxa, and associated functions of the gut microbiota between healthy subjects and early RA patients highlight the involvement of the gut microbiome in the early stages of RA.

## 1. Introduction

Rheumatoid arthritis (RA) is a representative autoimmune disorder characterized by inflammation in the synovial membrane of joints that spreads to inflammation in the surrounding cartilage and bone, resulting in joint destruction and deformation [1]. In addition to joint symptoms, it is a systemic disorder that causes extra-articular symptoms such as hematological abnormalities, dry eye syndrome, pulmonary fibrosis, vasculitis, and rheumatoid nodules [2].

In most patients, RA follows a continuum because occurrence begins with a susceptibility stage a few years before the clinical disease becomes apparent, after which the disease proceeds through preclinical RA followed by articular inflammation during the symptomatic stage [1]. Like other autoimmune diseases, such as lupus erythematosus, type 1 diabetes, and Sjogren’s syndrome, RA occurs more frequently in women [3,4]. The etiology is not clear, but both genetic and environmental causes are considered risk factors [1]. Recently, microorganisms have arisen as a risk factor among various environmental factors [5]. Animal studies aimed at deciphering the pathogenic process of several bacteria suggested a possible association between microorganisms and RA. In mice, host intestinal Th17 lymphocytes are induced by commensal bacteria, segmented filamentous bacteria (SFB) [6]. The introduction of SFB into germ-free mice caused induction of lamina propria Th17 cells, production of autoantibodies, and arthritis [6]. Moreover, Th17 cells were increased in the large intestine of SKG mice treated with *Prevotella copri* from the microbiota colonizing RA patients [7]. Clinical studies analyzing the fecal microbiome of RA patients are also on the rise. Microbial diversity was found to be reduced in RA patients in various studies, resulting in gut microbiota dysbiosis [8]. Picchianti et al. observed no difference at the phylum level between patients and healthy subjects, but members of the Bacilli class and Lactobacillales order were increased, and the *Faecalibacterium prausnitzii* species was decreased in RA patients not receiving therapy [9]. Studies on Chinese patients reported that *Lactobacillus* was increased in cases of very active RA [8], and in new onset RA patients, *Prevotella copri* was more abundant than in healthy subjects but less abundant in established RA cases [10]. In other research, *Prevotella copri* was more enriched in individuals at risk for rheumatoid arthritis compared with their first degree relatives [11].

Few studies regarding the fecal microbiome of patients with preclinical RA have been reported, and likewise for studies targeting RA female patients. Thus, in the present work we investigated the fecal microbiome of early RA female patients not receiving disease-modifying antirheumatic drugs (DMARDs), and investigated gut microbial functions to explore their association with RA disease activity.

## 2. Materials and Methods

### 2.1. Research Subjects

This study was approved by the ethics committee of Seoul St. Mary’s Hospital, Catholic University of Korea (KC17TNSI0570). Informed consent was obtained from all the study participants. Female healthy control subjects (CT, *n =* 25) and female patients with early RA (ER, *n =* 29) were enrolled in this study. Patients with early RA included patients with preclinical RA (PC, *n =* 17) and patients with clinically apparent RA (ST, *n =* 12). Patients with preclinical RA were defined as those at increased risk of developing RA. They have systemic autoimmunity associated with RA more than 3 times the reference value of rheumatoid factor (RF) and/or anti-citrullinated protein antibodies (ACPAs) with arthralgia without synovitis. Presence of synovitis was determined by physical examination and musculoskeletal ultrasound. Clinically apparent RA was defined as the presence of overt synovitis less than 6 months fulfilling the 2010 American College of Rheumatology/European League Against Rheumatism classification criteria for RA [12]. Patients with early RA were not treated with conventional disease-modifying antirheumatic drugs (DMARDs) other than hydroxychloroquine, biological DMARDs, or prednisolone equivalent >7.5 mg per day (nonsteroidal anti-inflammatory drugs were allowed). Enrolled patients did not have interstitial lung disease and other extra-articular manifestations. Comorbidities such as diabetes, hypertension, hyperlipidemia, and osteoporosis were not significantly different between preclinical RA and early RA patients (all *p >* 0.05, Fisher’s exact test). People using antibiotics, probiotics, or prebiotics at the time of sample collection were excluded from subject selection. Clinical information of patients related to RA is shown in Table 1.

### 2.2. Sample Collection and Next Generation Sequencing

Fecal samples were collected in a provided plastic container and sent immediately to the experimental site on ice. Within 12 h of arrival, samples were frozen and stored at −70 °C. Bacterial genomic DNA was extracted from stool samples from each subject using a QIAamp DNA Stool Mini Kit (Qiagen, Hilden, Germany) following the instructions of the manufacturer, followed by bead beating on a TissueLyser system (Qiagen). Bacterial genomic DNA was quantified using a Qubit 3.0 Fluorometer (Thermo Fisher Scientific, Waltham, MA, USA). For sequencing, 16S rRNA gene amplification and index PCR were conducted following the Illumina 16S Metagenomic Sequencing Library preparation guide (Illumina, San Diego, CA, USA). The 16S sequence was amplified using forward primer (5’-TCGTCGGCAGCGTCAGATGTGTATAAGAGACAGCCTACGGGNGGCWGCAG-3’), and reverse primer (5’-GTCTCGTGGGCTCGGAGATGTGTATAAGAGACAGGACTACHVGGGTATCTAATCC-3’). For indexing prior to DNA amplification, Nextera XT Index 1 and 2 Primers from a Nextera XT Index kit (Illumina) were used. Each PCR product was purified using AMPure XP beads (Beckman Coulter, Pasadena, CA, USA). DNA sequencing was conducted in using the paired end method (300bpx2) with an Illumina Miseq instrument according to the Illumina protocol.

### 2.3. Bioinformatics Analysis

Raw 16S rRNA gene sequences were filtered PhiX reads and chimeric sequences and merged to single end sequences using the DADA2 plugin in the qiime2-2018.11 platform [13]. Sample metadata used in the DADA2 plugin contained information such as age, sex, and various clinical parameters for categorical and numerical formatting. Filter parameter for trimming and truncating using the DADA2 plugin were 0 and 280 to remove low-quality regions of sequences. A feature table generation was then conducted using ‘qiime feature-table summarize’, and ‘qiime feature-table tabulate-seqs’ plugins in qiime2-2018.11. The phylogenetic tree construction was performed by ‘qiime phylogeny align-to-tree-mafft-fasttree’ using qiime2-2018.11. Alpha and beta diversity analyses were conducted using ‘core-metrics-phylogenetic’ plugin in qiime2-2018.11 and calypso with a depth of 5307 reads [14]. Group significance test and visualization of beta diversity parameters were carried out with the ‘qiime diversity beta-group-significance’ plugin and the ‘qiime emperor plot’ plugin in qiime2-2018.11. The ‘qiime feature-classifier’ plugin was used for taxonomic analysis to explore the taxonomic composition of the samples and relate the results to sample metadata. Featured taxa selection by linear discriminant analysis (LDA) effect size (LEfSe) and random forest prediction were executed using LEfSe and calypso [15]. Microbial function analysis was performed using phylogenetic investigation of community by reconstruction of unobserved States (PICRUSt) in qiime1.9.1. [16].

### 2.4. Statistical Analysis

The number of read in the feature table/operational taxonomic unit (OTU) table was normalized based on the total sum method to generate relative abundance. Non-parametric statistical analyses including Wilcoxon rank-sum tests and Kruskal–Wallis tests were carried out to compare the relative abundance of taxa and alpha diversity of each group. PERMANOVA tests were used to compare beta diversity between each group. Spearman correlation test were executed to analyze correlations between relative abundance and clinical parameter value. All statistical analysis was conducted using Prism8 (GraphPad Software, Inc., San Diego, CA, USA) and qiime2-2018.11.

## 3. Results

### 3.1. Differences in Gut Microbial Diversity between RA Patients and Healthy Subjects

**Figure 1:**
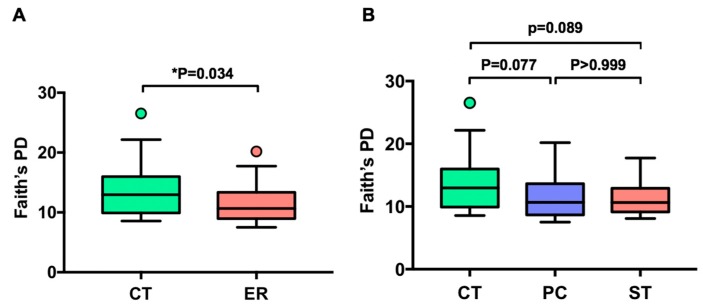

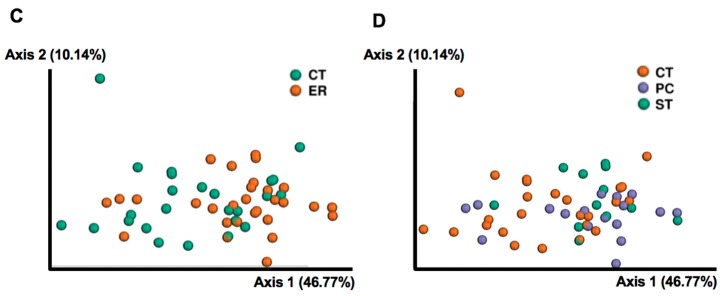
Comparison of bacterial diversity and communities between early rheumatoid arthritis patients (ER) and healthy controls (CT). (**A**,**B**) Faith’s phylogenetic diversity comparisons were performed for ER and CT groups using Wilcoxon rank-sum tests. Comparisons among CT, PC, and ST groups were performed using Kruskal–Wallis tests. Lines inside the box represent the median, while whiskers represent the lowest and highest values within the 1.5 interquartile range (IQR). Outliers and individual sample values are shown as dots. (**C**,**D**) Principal coordinate plot based on the weighted-UniFrac distance matrix.

To investigate whether RA is associated with altered microbial diversity, we sequenced fecal samples from 25 healthy subjects and 29 early RA female patients (Table 1) and analyzed the data using qiime2-2018.11. After filtering, merging and removing chimera and PhiX sequences, 543,259 reads were clustered into OTUs based on 97% sequence similarity, with a mean of 10,060 reads across the 54 samples. The phylogenetic diversity of the gut microbiota of ER patients was significantly different from that of healthy subjects (*p* < 0.05 for Faith’s phylogenetic diversity (PD), Figure 1A). However, when ER patients were subdivided into two patient groups with PC and ST, there were no differences between PC and ST patients (*p* > 0.999 for Faith’s PD, Figure 1B). Shannon diversity index, which reflects both evenness and richness, presented a similar declining trend in terms of phylogenetic differences between different ER patients but this was not significant (*p* = 0.157, Figure S1A,B). The richness of the gut microbiota of ER patients was not significantly different from that of the CT group (*p* = 0.1547, Figure S1C,D). Weighted UniFrac distance-based microbiota structure analysis revealed significant differences in microbial community between ER patients and healthy subjects (*p* = 0.008, PERMANOVA, Figure 1C). The community in the PC patients’ microbiota was similar to that in ST patients (*p* = 0.723, PERMANOVA, Figure 1D), and both the PC and ST groups differed from healthy subjects (*p* = 0.0249 and *p* = 0.040, PERMANOVA, Figure 1D).

### 3.2. Featured Microbial Taxa in ER Patients

**Figure 2:**
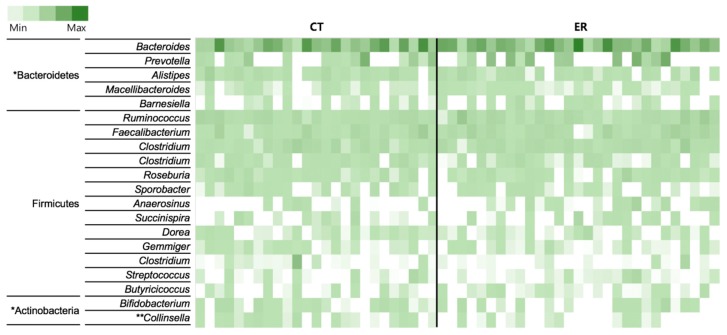
Heat map of the 20 most abundant genera in each sample. The color intensity in each cell indicates the proportion of a given genus in a sample. Significantly abundant taxa in either of the two groups (* *p* < 0.05, Wilcoxon rank-sum test; ** *p* < 0.01, Wilcoxon rank-sum test) are included.

We analyzed the bacterial composition of each group at genus and OTU levels as shown in Figure 2, Figure S2, and Figure S3, and using Wilcoxon rank-sum tests, limited the analyses to taxa with a prevalence >30%, to identify taxa differing in abundance between the two groups at phylum, class, order, family, and genus levels especially within the top 20 taxa at the genus level (Table 2). We identified 11 differentially abundant taxa at a false discovery rate of 10%, with three taxa enriched in ER and eight enriched in CT. Within the phylum Bacteroidetes, the class Bacteroidia and the order Bacteroidales were more abundant in ER than in CT (Table 2). The Prevotella genus in the Bacteroidetes phylum, known to be induced in RA, was highly abundant in ER (Figure S2A). Among the Actinobacteria phylum, the *Collinsella* genus showed significant difference between CT and ER for the Coriobacteriia class, Coriobacteriales order and Coriobacteriaceae family (Table 2). In addition to the differnce in the relative abundance of the *Collinsella* genus, the prevalence also decreased from 76% in CT to 41% in ER patients (Table 2).

**Figure 3:**
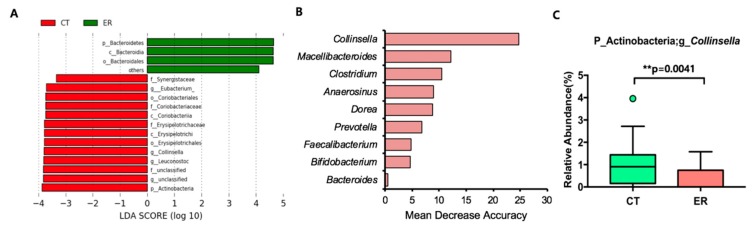
Featured taxa based on LDA effect size (LEfSe) analysis and random forest predictive analysis of CT and ER groups. (**A**) Selected taxa based on LEfSe analysis. (**B**) Selected taxa based on random forest analysis of the top 20 abundant genera. (**C**) Relative abundance of the genus Collinsella.

To confirm the differences in bacteria ecology between ER and CT groups, LEfSe and random forest prediction were performed. Similar to the rank-sum test results, the phyla Actinobacteria and Bacteroidetes and the genus Collinsella with fewer taxa yielded different LDA scores (Figure 3A). The Collinsella genus was the most important taxa among the top 20 genera and the Prevotella genus was important for classifying patients by random forest prediction analysis (Figure 3B,C, and Figure S2A). Bifidobacterium, another genus within the phylum Actinobacteria, was also important according to random forest prediction, and was reduced in ER patients compared with the CT group (Figure 3B and S2B). In the rank-sum test and LEfSe analysis, no taxa were found to be differentially abundant between PC and ST groups.

### 3.3. Microbial Function Analysis

**Figure 4:**
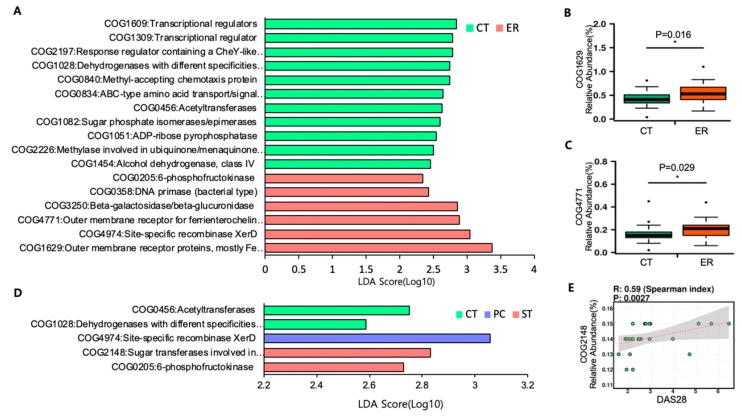
Featured COGs by PICRUSt. (**A**) Differentially abundant COGs between ER and CT groups based on LEfSe analysis. (**B**,**C**) The relative abundance of COG1629 and 4771. (**D**) Differentially abundant COGs among CT, PC, and ST groups based on LEfSe analysis. (**E**) Spearman correlation analysis of COG2148 and DAS28.

PICRUSt was used to analyze functional genes of microbial communities in ER patients. Using function predictions based on clusters of orthologous group (COG) analysis, we found 17 significantly different functional COGs between ER and CT groups (Figure 4A). The function specifically associated with the microbiota in ER patients included notable enrichment of iron transport and absorption, as examplified by COG 1629 and 4771, whereas functions related to ubiquinone (coenzyme Q10)/menaquinone(vitamin K2) biosynthesis such as COG 2226 were enriched in the CT group (Figure 4A–C). Although there were no significant differences in microbial structure, we searched microbial functions distinct in PC or ST among ER patients. Interestingly, COG 2148, sugar transferases involved in LPS synthesis, was abundant in the ST group among ER patients (Figure 4D) and was positively correlated with the DAS28 index representing the degree of disease activity of RA (Figure 4E).

## 4. Discussion

This study investigated the differences in diversity and changes in specific bacterial taxa in gut microbiomes associated with early RA, as well as differences in microbial functions among patients at preclinical and symptomatic stages based on the clinical parameters. Scher et al. (2013) compared the gut microbiota of new onset RA patients and healthy control subjects and noted significant differences in diversity and composition [10]. In the present study, alpha diversity was reduced in RA patients as reported in the previous study. However, while a significant increase in the genus *Prevotella* was observed previously in RA onset patients, there was only a slight increase in the present study. Random forest analysis showed that *Prevotella* is an important genus for classifying ER patients. Presumably, the results for new onset RA patients were not consistent between studies due to significant differences in subject composition. RA is more prevalent in females, hence all our subjects were female, whereas the preceding study was composed of 75% women and 25% men. There were also differences in disease activity parameters, such as disease activity score 28 (DAS28), C-reactive protein (CRP), and erythrocyte sedimentation rate (ESR). DAS28 is a combined measurement of the number of swollen and tender joints among the 28 body joints and other laboratory data. It is classified as low when <3.2 and moderate/high when ≥3.2 [17]. The average DAS28 score of early RA patients in the present study was low (2.92) while that of the new onset RA group in the previous study was moderate/high (4.8). Hence, early RA patients in the present study appear to be closer to the actual early stage than new onset RA in the former study. In addition, CRP was 5.9 mg/L in the present study, significantly lower than 20.6 mg/L measured in the preceding study, and ESR was also lower in the present work (16.55 mm/h) than in the former study (34.6 mm/h). Based on all available clinical information, it appears that the present study was conducted at an earlier stage than the previous study, and may further indicate an association between the microbiome and early RA etiology.

Few reports have examined the gut microbiota of RA patients at all stages from early onset to the established. Studies showed that *Lactobacillus salivarius*, *Fretibacterium*, *Selemonas*, *Bacilli*, and other groups are increased in RA, while *Faecalibacterium*, *Blautiaccoids,* and *Flavobacterium* are decreased [8,9]. The genus *Collinsella*, which appeared to be decreased in ER versus CT in the present study, was revealed as a potential pathogenic microbe by Chen et al. [18]. These differences between the two studies may be to due to differences between subjects; the present study was carried out on drug-naïve early RA female patients whereas the previous study was conducted on RA patients including those treated with various medications. It is not clear whether the intestinal microbial conditions at the time of therapeutic administration are more dependent on disease or drug effects. Several studies demonstrated that both *Collinsella* and *Bifidobacterium* genera within the Actinobacteria phylum can have a positive effect on the gut health, such as the adjustment of host bile acids to regulate the pathogenicity and virulence of enteric pathogens [19]. Additionally, in fecal sample from irritable bowel syndrome (IBS) patients, a representative intestinal disorder, the genus *Collinsella* was reduced compared with healthy controls [20]. In this context, the reduction in Actinobacteria and *Collinsella* in ER patients, and the random forest results showing that *Collinsella* and *Bifidobacterium* are important genera for classifying ER patients demonstrate the association between microbiome and RA, a systemic inflammatory disease. Intriguingly, the Bacteroidales order, which is reported to be enriched in gut microbiota of patients with melanoma, was enriched in ER in the present work [21]. Moreover, Bifidobacterium was decreased in microbiome of patients with melanoma [22]. Despite the differences in the disease, similar trends in microbiome changes were observed in patients with RA and melanoma, health-threatening diseases.

The bacterial composition in RA patients can differ depending on various factors such as the experimental site, disease status, medication given to patients, and sex [4,9,23,24]. However, in most studies, including our present work, diversity indices related to phylogenetic diversity, species richness, and evenness tend to be reduced in RA patients compared with healthy subjects [8,14]. The apparent decrease in diversity in RA patients, including early stage RA patients, may be an indication of the association between the etiology of RA and the microbiota.

In the present study, we also discovered altered microbial functions in patients with early RA compared with healthy subjects using PICRUSt based on COG analysis. In ER patients, iron absorption-related genes such as those in COG 1629 and 4771 were enriched. COG1629 and 4771 are gene clusters associated with outer membrane receptor proteins that bind iron-chelating siderophores to facilitate bacterial absorption of iron. This is likely to result in frequent anemia in RA patients, and consequent alterations to the gut microbiota [25,26]. Intriguingly, although PC and ST patients did not show any difference in microbiome structure, COG2148 (sugar transferases involved in LPS synthesis) was enriched only in ST patients. LPS is a gram-negative bacteria-derived inflammatory substance that can spread all throughout the host through blood vessels; hence it can be considered in association with RA, a systemic inflammatory disease. COG2148 was also positively correlated with DAS28, a disease activity parameter of RA, suggesting that the microbiome is related to the flare of RA. While gram-negative bacteria-related COG was enriched in the most active group among ER patients, so was COG2226 encoding methylase involved in ubiquinone (Coenzyme Q10)/menaquinone (Vitamin K2), a known gram-positive bacteria-related gene cluster enriched in the CT group [27]. In this context, it is possible that LPS derived from gram-negative bacteria, which is generally enhanced in ST versus CT groups, may affect the pathogenesis of early RA or the onset of disease.

The present work adds support to the result of preceding studies and further demonstrates the involvement of the gut microbiota in early RA. Specifically, the present work demonstrates alterations in the microbial community, including microbial functions. Further investigation of the differences in microbiota between PC and ST patients is needed to clarify the role of the microbiome in RA development. Future studies on larger and subdivided cohorts incorporating other omics technologies such as metatranscriptomics and metabolomics could illuminate the causal and effect relationships between the gut microbiota and RA.

## Figures and Tables

**Table 1:** Clinical features of early rheumatoid arthritis patients and healthy controls.

	ER			CT
	PC	ST	Total	
N	17	12	29	25
Age	47.71 ± 6.57	44.33 ± 14.65	46.31 ± 10.58	42.32 ± 12.46
Sex (F/M)	17/0	12/0	29/0	25/0
** DAS28	2.26 ± 0.43	3.84 ± 1.52	2.92 ± 1.28	-
** CRP (mg/dL)	0.11 ± 0.19	1.28 ± 1.77	0.59 ± 1.26	-
** ESR (mm/h)	9.06 ± 5.83	27.17 ± 17.78	16.55 ± 15.03	-
RF (IU/mL)	188.20 ± 285.93	288.10 ± 245.74	203.90 ± 266.85	-
ACCP (CU)	1048.00 ± 2815.19	765.30 ± 705.84	942.10 ± 2239.86	-

The values are presented as the mean ± standard deviation or frequency. Comparisons of parameters between early RA (ER) and healthy controls (CT) or patients with preclinical RA (PC) and patients with clinically apparent RA (ST) groups were statistically assessed through Wilcoxon’s rank-sum tests. The double asterisk indicates statistically significant different of each value between PC and ST with *p*-value less than 0.01. DAS28: disease activity score 28 joints, CRP: C-reactive protein, ESR: erythrocyte sedimentation rate, RF: Rheumatoid factor, ACCP: anti-cyclic citrullinated peptide.

**Table 2:** Differentially enriched taxa in early rheumatoid patients and healthy controls showing the top 20 taxa at each level.

	Taxa	*p*-value	FDR	CT Mean (%)	ER Mean (%)	Fold Change	CT Prevalence (%)	ER Prevalence (%)
Enriched taxa in ER								
Phylum	Bacteroidetes	* 0.011	**0.091**	34.78	43.07	1.24	100	100
Class	Bacteroidia	* 0.014	**0.089**	34.67	43.03	1.24	100	100
Order	Bacteroidales	* 0.014	**0.093**	34.67	43.03	1.24	100	100
Enriched taxa in CT								
Phylum	Actinobacteria	* 0.014	**0.091**	2.85	1.52	0.54	92	69
Class	Erysipelotrichi	** 0.0027	**0.028**	1.44	0.65	0.45	96	72
Class	Coriobacteriia	** 0.0029	**0.028**	1.35	0.48	0.36	80	48
Order	Erysipelotrichales	** 0.0027	**0.029**	1.44	0.65	0.45	96	72
Order	Coriobacteriales	** 0.0029	**0.029**	1.35	0.48	0.36	80	48
Family	Erysipelotrichaceae	** 0.0027	**0.029**	1.44	0.65	0.45	96	72
Family	Coriobacteriaceae	** 0.0029	**0.029**	1.35	0.48	0.36	80	48
Genus	*Collinsella*	** 0.0041	**0.082**	1.00	0.36	0.36	76	41

Comparisons were analyzed using Wilcoxon rank-sum tests. The single- and double-asterisk indicates statistically significant different of each value between CT and ER with *p*-value less than 0.05 and 0.01. Bold values indicate a significant comparison with a false discovery rate (FDR) <0.1.

## Supplementary Materials

The following are available online at https://www.mdpi.com/2077-0383/8/5/693/s1, Figure S1: Comparison of alpha diversity in RA patients and healthy controls. Figure S2: The relative abundance of specific taxa. Figure S3: Microbial composition of the top 30 OTUs in CT and ER groups. Figure S4: Microbial composition of the top 30 OTUs in CT, PC, and ST.
