# Supplementary material for: Gut Microbial Composition and Function Are Altered in Patients with Early Rheumatoid Arthritis
Source: J Clin Med. 2019 May 16;8(5):693. doi: 10.3390/jcm8050693 (PMC6572219; doi:10.3390/jcm8050693)
Supplement: Supplementary file 1 [file jcm-08-00693-s001.pdf]

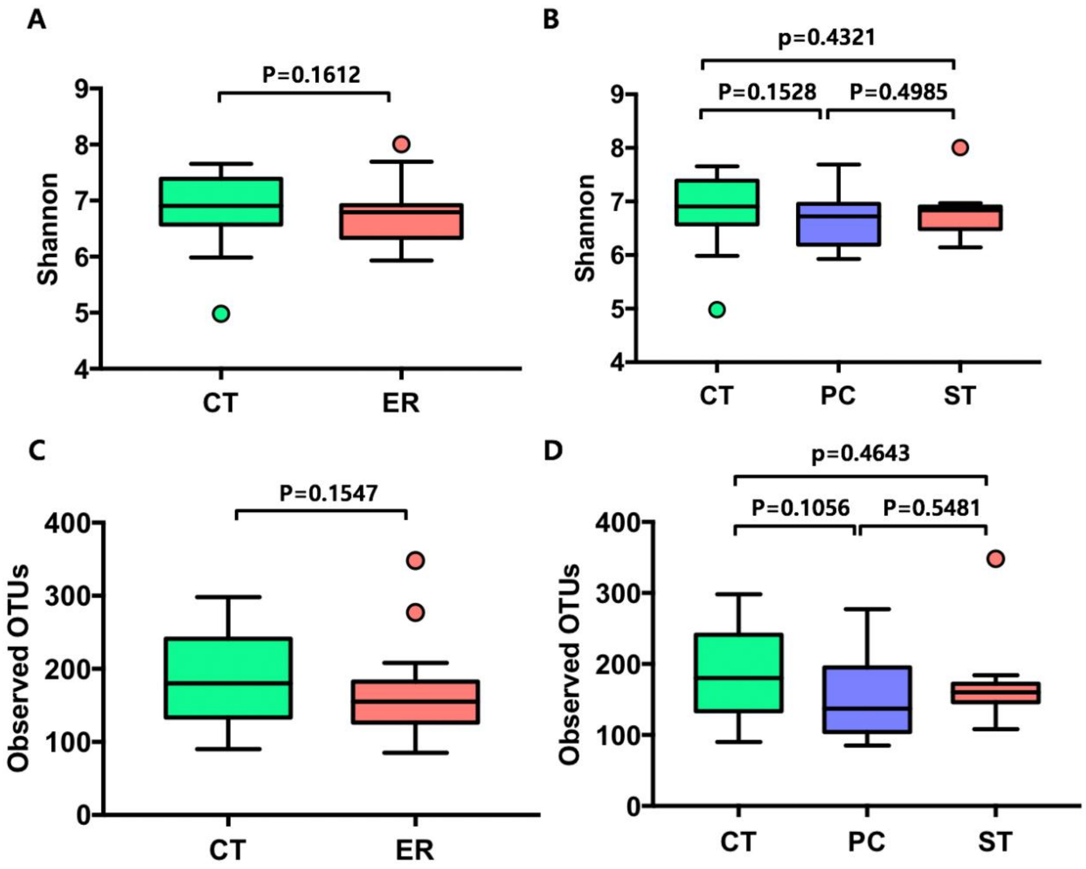

Figure S1. Comparison of alpha diversity in RA patients and healthy controls. (A, B) Shannon index comparisons were performed for ER and CT using Wilcoxon rank sum tests. Comparison among CT, PC, and ST were performed using Kruskal Wallis tests. (C, D) Comparison of the number of Observed OTUs in ER and CT groups using Wilcoxon rank-sum tests. Comparisons among CT, PC, and ST groups were performed using Kruskal Wallis tests.

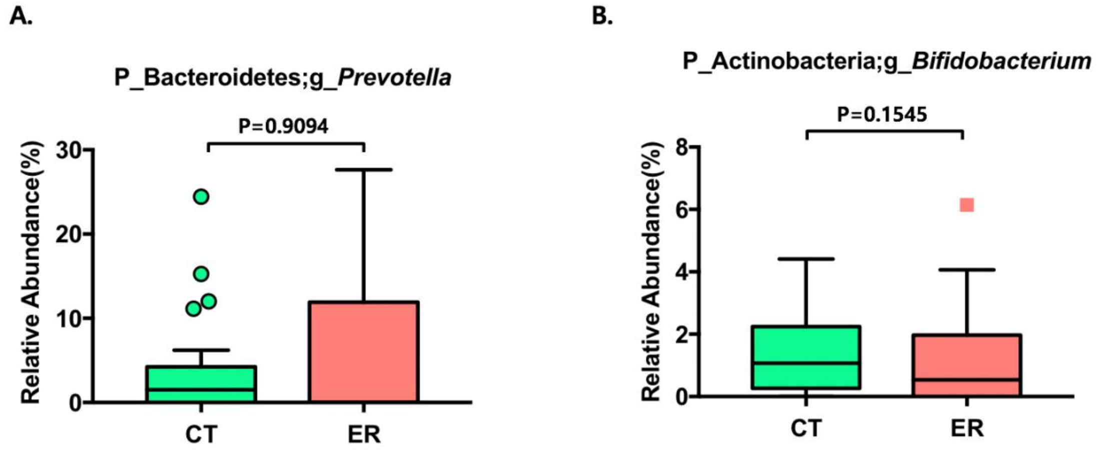

Figure S2. The relative abundance of specific taxa. (A) Relative abundance of the *Prevotella* genus. (B) Relative abundance of the *Bifidobacterium* genus. Comparisons were performed using Wilcoxon rank-sum tests.

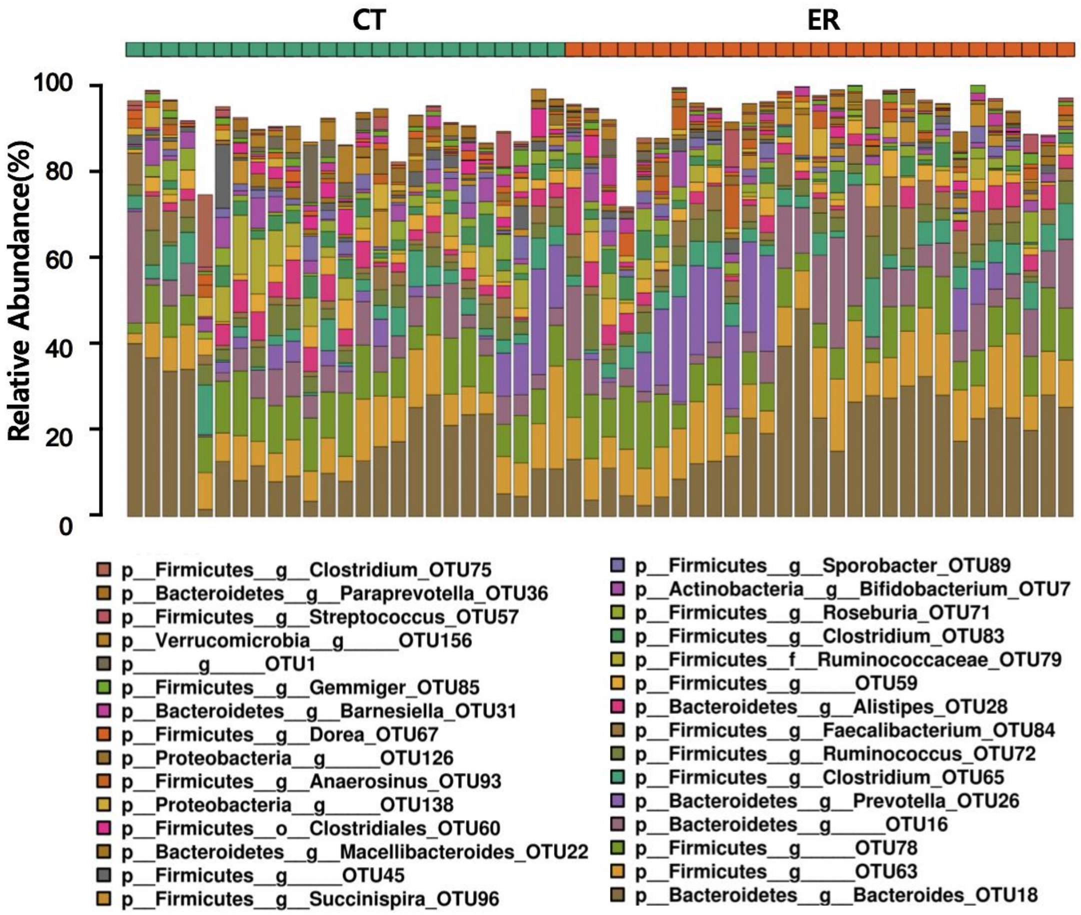

Figure S3. Microbial composition of the top 30 OTUs in CT and ER groups.

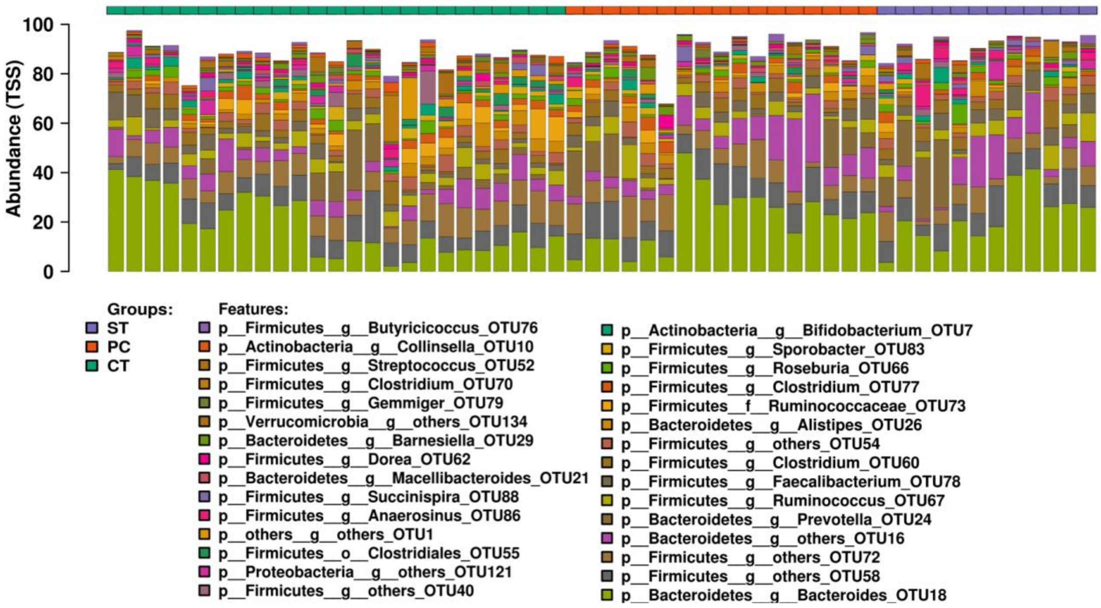

Figure S4. Microbial composition of the top 30 OTUs in CT, PC, and ST groups.
